# Ultraviolet Laser Lithography of Titania Photonic Crystals for Terahertz-Wave Modulation

**DOI:** 10.3390/ma11050835

**Published:** 2018-05-18

**Authors:** Soshu Kirihara, Koki Nonaka, Shoichiro Kisanuki, Hirotoshi Nozaki, Keito Sakaguchi

**Affiliations:** 1 Joining and Welding Research Institute, Osaka University, 11-1 Mihogaoka Ibaraki, Osaka 567-0047, Japan; 2 Graduate School of Engineering, Osaka University, 2-1 Yamadaoka Suita, Osaka 565-0871, Japan; nonaka@jwri.osaka-u.ac.jp (K.N.); kisanuki@jwri.osaka-u.ac.jp (S.K.); nozaki@jwri.osaka-u.ac.jp (H.N.); sakaguchi@jwri.osaka-u.ac.jp (K.S.)

**Keywords:** photonic crystal, terahertz wave modulation, additive manufacturing, ultraviolet laser lithography, titanium dioxide, nanoparticles paste

## Abstract

Three-dimensional (3D) microphotonic crystals with a diamond structure composed of titania microlattices were fabricated using ultraviolet laser lithography, and the bandgap properties in the terahertz (THz) electromagnetic-wave frequency region were investigated. An acrylic resin paste with titania fine particle dispersions was used as the raw material for additive manufacturing. By scanning a spread paste surface with an ultraviolet laser beam, two-dimensional solid patterns were dewaxed and sintered. Subsequently, 3D structures with a relative density of 97% were created via layer lamination and joining. A titania diamond lattice with a lattice constant density of 240 µm was obtained. The properties of the electromagnetic wave were measured using a THz time-domain spectrometer. In the transmission spectra for the Γ-X <100> direction, a forbidden band was observed from 0.26 THz to 0.44 THz. The frequency range of the bandgap agreed well with calculated results obtained using the plane–wave expansion method. Additionally, results of a simulation via transmission-line modeling indicated that a localized mode can be obtained by introducing a plane defect between twinned diamond lattice structures.

## 1. Introduction

Photonic crystals with periodic-arrangement structures as a dielectric medium can prohibit electromagnetic-wave propagation caused by Bragg reflection, and exhibit forbidden gaps in the transmission spectra [1,2]. Bandgap profiles are controlled arbitrarily via theoretical modulations of cavity introductions into the dielectric arrangements. Electromagnetic waves with typical wavelengths comparable to the cavity sizes are resonated in artificial crystal defects, and electromagnetic energy localizations and amplifications can form permission modes of transmission peaks in photonic bandgaps.

Three-dimensional (3D) photonic crystals with diamond structures are regarded as ideal dielectric patterns to exhibit complete bandgaps, prohibiting incident electromagnetic waves from all directions [3]. Diamond photonic crystals can be applied to resonators, wavelength filters, directional antennas, etc. [4,5]. Our previous studies employed alumina photonic crystals comprising complicated diamond microlattices with four coordination numbers, which were fabricated via stereolithographic additive manufacturing and powder sintering [6,7]. Through computer-aided design, manufacturing, and evaluation, micro-defects in the form of point- or plane-shaped cavities were introduced to control the terahertz (THz) waves with micrometer-order wavelengths [8,9]. THz waves are expected to detect micro-cracks in material surfaces and structural defects in electrical circuits via fine wave interference, and are used to analyze cancer cells in human skin and toxic bacteria in natural foods through high-frequency excitations [10,11,12,13,14].

In our evolved stereolithography method, an ultraviolet laser beam was scanned across a spread paste of photo-curing resin containing fine ceramic particles to create two-dimensional (2D) cross-sectional layers, and 3D structures with geometric patterns were formed via layer lamination and joining though photopolymerization. The obtained composite precursors were dewaxed and sintered to obtain dense components without structural deformation. Linear shrinkage corresponding to the particle dispersion amounts was controlled along the optimized heat-treatment patterns. Recently, we discovered that an ultraviolet laser beam with a short wavelength and high power could propagate between dispersed fine particles, and remove resin menstruum via heat decomposition. Through the optimization of laser-beam spot size, scanning speed, and irradiation power, we sintered the remaining ceramic powder and increased the relative density.

Titania was selected as the dielectric material of the microlattice, and the structural dimensions of the diamond structures were designed to open the complete bandgap in the THz frequency range. The titania microphotonic crystals were fabricated via ultraviolet laser lithography. The microstructures of the ceramic lattices were observed to optimize the laser-beam conditions for increasing the relative densities. The transmission spectra for typical crystal directions were measured via time–domain spectroscopy. Opened gap frequencies were compared with an electromagnetic band diagram, theoretically calculated using Maxwell’s equations. The localized mode formation in the plane defects between twinned diamond lattices was visualized using numerical simulations. The peak frequencies of permission modes were compared with the inner profiles of the measured bandgaps.

## 2. Design and Calculation

The band profiles of a photonic crystal can be modulated according to periodic pattern and dielectric property. A diamond structure with a higher dielectric constant and a shorter lattice constant opens a wider bandgap in a higher frequency range. A volume fraction of a dielectric material to an air gap should be optimized via systematic adjustments of an aspect ratio, i.e., a ratio of lattice diameter to lattice length, to obtain perfect photonic bandgaps opened for all crystal directions.

**Figure 1:**
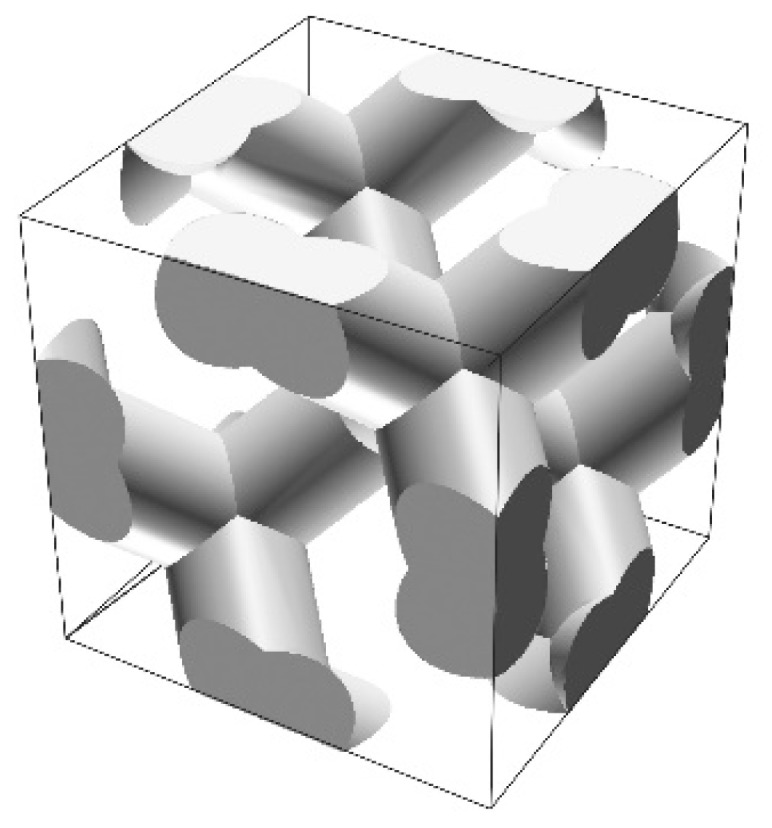
Designed computer graphic model of a unit cell in a photonic crystal. A diamond structure was composed of four coordination lattices with an aspect ratio of 1.5. The volume fraction of the dielectric material was chosen to be 33%.

**Figure 2:**
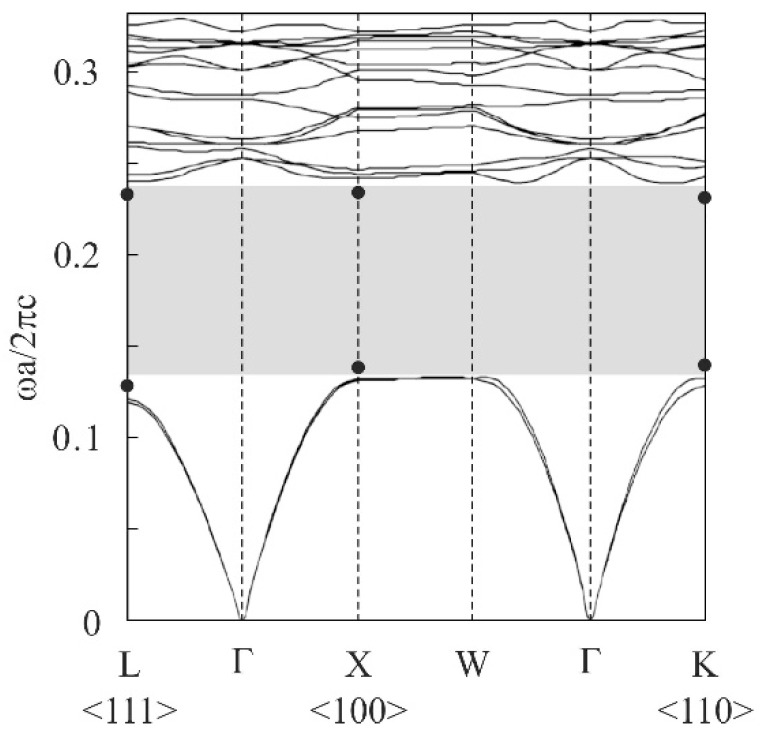
Electromagnetic band diagram calculated using the PWE method. The dielectric constant and the volume fraction of the lattice were 100% and 33%, respectively. A gray band shows a perfect photonic bandgap. Solid circles indicate measured bandgap edges.

A unit cell of the diamond structure was designed, as shown in Figure 1, by using a computer graphics software (T3 Japan: Think Design). To obtain an ideal bandgap, an aspect ratio and a volume fraction of the dielectric lattices were adjusted to 1.5% and 33%, respectively. The electromagnetic band diagram of the designed diamond structure was calculated along the symmetry lines in a Brillouin zone via the plane-wave-expansion (PWE) method (BandSOLVE: CYBERNET, Tokyo, Japan), as shown in Figure 2 [15]. A dielectric constant of 100 was selected as the material parameter of the titania lattices. Plane waves of 124 in number propagated into the imaginarily limitless periodic arrangement of dielectric lattices. By modulating the lattice aspect ratio from 1.0 to 2.0, the widest perfect bandgap of 1.5 was obtained.

**Figure 3:**
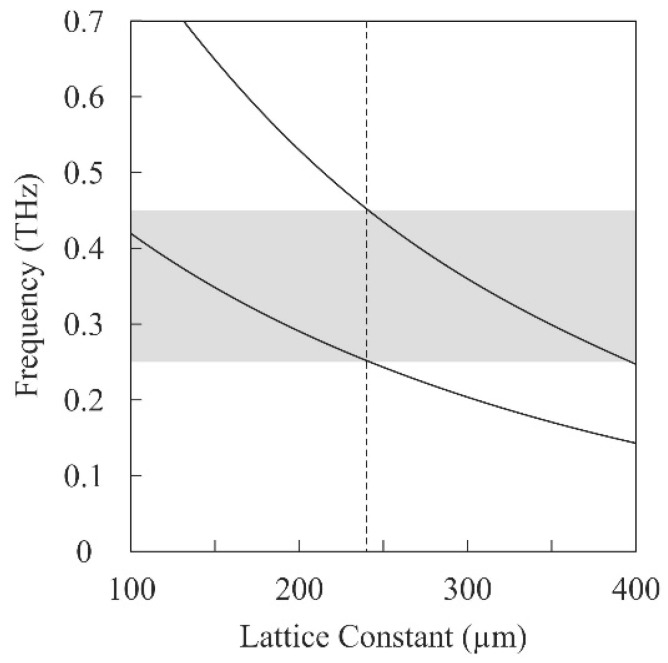
Frequency variations of the higher and lower edges at perfect photonic bandgaps exhibited by the diamond structures calculated using the electromagnetic band diagram shown in Figure 2. The dotted line indicates a minimum lattice constant of 240 µm to open the bandgap ranging from 0.25 THz to 0.45 THz, indicated by the gray band.

Figure 3 shows the frequency variations of the perfect bandgaps according to the lattice constants. The upper and lower solid lines indicate the higher and lower edges of the bandgap frequencies, respectively. The gray band from 0.25 THz to 0.45 THz indicates the intended frequency range for the photonic bandgap, and the dotted line at 240 μm determined the preferable lattice constant of the diamond structure. The frequency ranges in the vicinity of 0.35 THz are used in novel imaging and inspection systems for package inspection, quality control and nondestructive testing [16]. Using ultraviolet laser lithography, a diamond structure with a minimum lattice constant of 240 µm was fabricated through 2D beam drawing with a spot size of 10 μm and 3D layer stacking with a lamination pitch of 10 μm.

**Figure 4:**
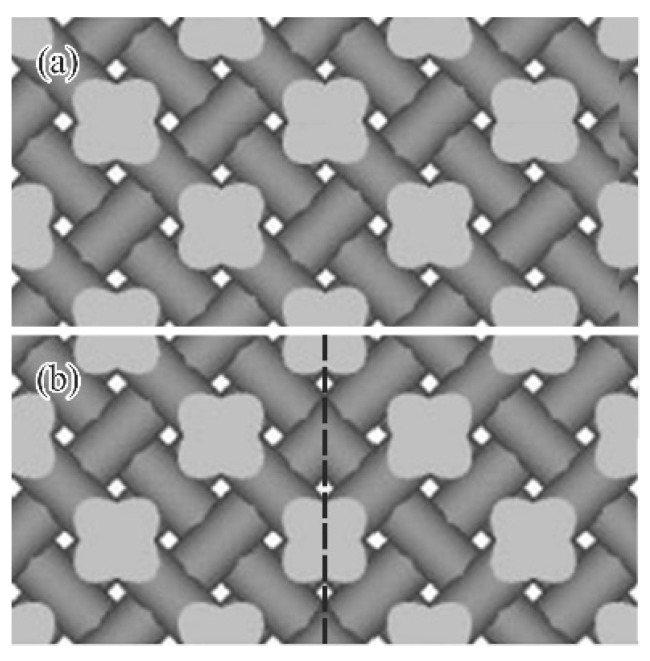
Designed graphic models of diamond photonic crystals composed of 20 × 20 × 3 unit cells (**a**) A stacking fault was introduced parallel to the (100) plane between mirror-symmetric diamond lattices of twinned crystals; (**b**) The dotted line indicates the plane defect.

The graphic data for the photonic crystal composed of perfect periodic lattices was finalized by connecting 20 × 20 × 4 unit cells, as shown in Figure 4a. The photonic crystal model of 4.8 × 4.8 × 0.96 mm^3^ was designed to fill an aperture having an opening diameter of 3 mm mounted on the THz-wave measurement system. The plane defect of the artificial stacking fault was introduced perpendicular to the (100) crystal plane between the twinned diamond lattices, as shown in Figure 4b.

## 3. Experimental Procedures

**Figure 5:**
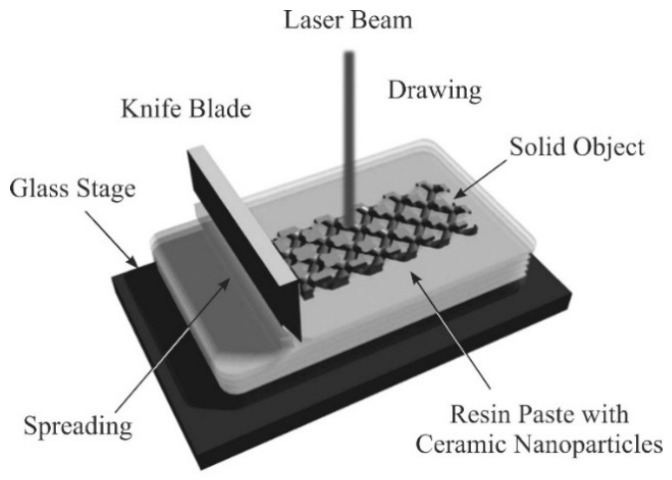
Schematic of the fabrication sequence for ultraviolet laser lithography as an additive manufacturing technology. Through laser-beam irradiation and ablation, cross-sectional patterns were dewaxed and sintered, and thin solid layers were laminated and joined.

The 3D models of the diamond photonic crystals were automatically converted into a stereolithographic file format with a modeling application (Magics: Materialise, Yokohama, Japan) through polyhedral approximations, and were sliced into a series of cross-sectional layers of the 2D patterns. The slicing pitch was defined at 10 µm. The numerical datasets were transferred into the ultraviolet laser lithography system (SZ-1000S: Shashin Kagaku, Kyoto, Japan) to create raster patterns for laser scanning. The additive manufacturing process is schematically illustrated in Figure 5. The titania particles, which had an average diameter of 270 nm, were dispersed into an acrylic resin (KC1278: JSR, Tokyo, Japan) at a volume fraction of 50%, without photo- or heat-curing. The obtained paste was spread on a flat glass stage using a mechanical knife edge. The thickness was controlled automatically according to the model slicing pitch. The paste surface was scanned at a speed ranging from 50–100 mm/s with a laser beam working at 355 nm to create cross-sectional planes through resin dewaxing and powder sintering, involving propagations and absorptions of the ultraviolet ray. The spot size was adjusted to 10 µm, and the irradiation power was varied from 600 mW to 700 mW. The laser conditions were indicated in Table 1. After the elevator stage moved down with respect to the layer thickness, the next cross section was formed and joined to the solid object. The 3D components were fabricated by stacking all the 2D layers.

The part accuracy and microstructural soundness of titania lattices were observed using digital optical microscopy (DOM) (VH-Z100: Keyence, Osaka, Japan), and scanning electron microscopy (SEM) (JSM 6060: JEOL, Tokyo, Japan). The relative density was measured via the Archimedes’ method, and the crystal phase was analyzed via X-ray diffraction (XRD) (Ultima: Rigaku, Tokyo, Japan). The dielectric constant and the loss were analyzed for the titania bulk by using a THz time-domain spectrometer (THz-TDS) (J-Spec 2010, Nippo Precision, Yamanashi, Japan). The bandgap exhibition and the localized mode formation were analyzed for the titania photonic crystals with and without the twinned defect in the diamond lattices. The intensity distributions of the THz waves in the periodically arranged lattices and the introduced plane defects were simulated and visualized, using the transmission-line modeling (TLM) method (CST Studio Suite: AET, Kawasaki, Japan) [17].

## 4. Results and Discussion

**Figure 6:**
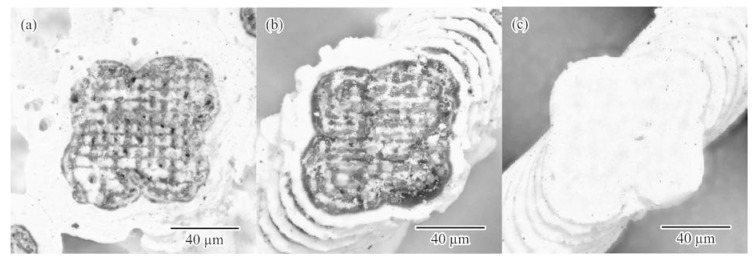
Titania photonic crystals with diamond structures fabricated via ultraviolet laser lithography. The laser-beam irradiation power and the scanning speed were optimized to increase the part accuracy and reduce the amount of remaining carbon. The ceramic lattices (**a**–**c**) were fabricated according to the laser conditions A, B and C, indicated in Table 1, respectively.

**Figure 7:**
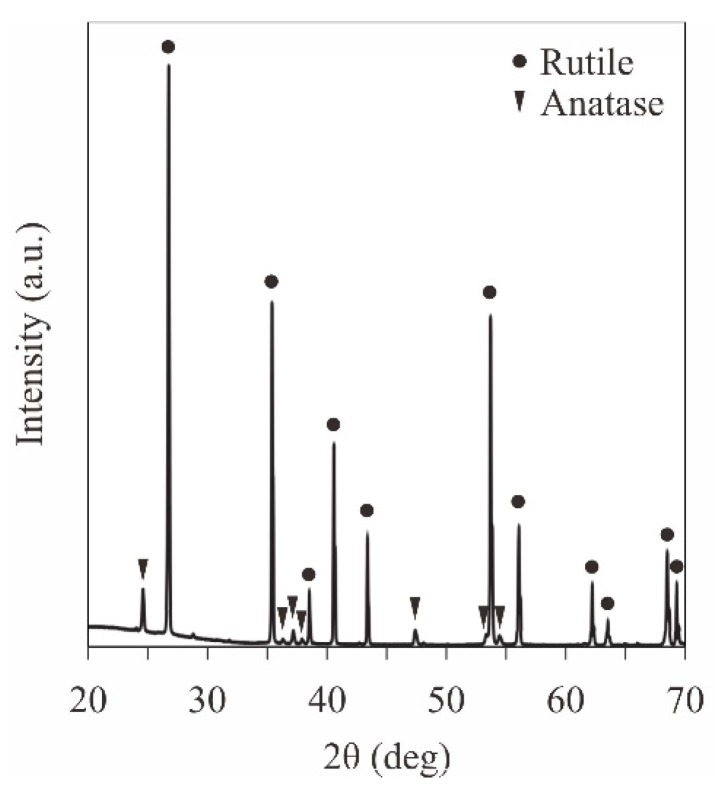
An XRD pattern of the titania lattice fabricated via ultra violet laser lithography, as shown in Figure 6c. The diffraction patterns, indicating dual phase of anatase and rutile, were observed without the residual carbon peaks.

The titania microlattices with diamond structures were fabricated through resin dewaxing and powder sintering together with ultraviolet laser lithography, as shown in Figure 6. The scanning speed and the irradiation power of the laser beam were adjusted to decrease the remaining carbon and increase the part accuracy of the ceramic components. The ceramic components Figure 6a–c were fabricated according to the laser conditions A, B and C, indicated in Table 1, respectively. In the case of Figure 6a, the spread resin paste containing nanoparticles was scanned at a moving speed of 50 mm/s with a laser beam at irradiation power of 600 mW. The titania particles were coagulated on the lattice surface though thermal conductions in the laser sintering. The residual carbon that accumulated during the deficient dewaxing of the acrylic resin solvent was observed as black areas in the lattice cross section. Subsequently, the laser scanning speed was increased to 100 mm/s to reduce the thermal conductions for the resin paste, and the coagulation of particles was prevented, as shown in Figure 6b. The part accuracy was measured to be ±5 µm using DOM. The residual carbon indicated by the black area was increased in the lattice simultaneously. Moreover, the laser-irradiation power was increased to 700 mW, and the black area disappeared in the lattice cross section, as shown in Figure 6c. An XRD pattern of the formed titania lattice is shown in Figure 7. The residual carbon peaks were not observed. The crystal structure of the titania was analyzed and revealed as a dual phase of anatase and rutile. The titania particles were considered to be heated in the vicinity of the temperature for the anatase-to-rutile phase transformation. A relative density of the ceramic components of 97% was reached in the Archimedes’ measurement.

**Figure 8:**
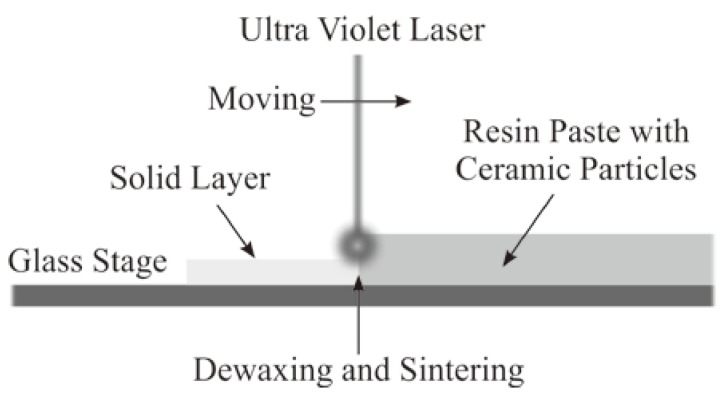
Cross-sectional schematic of the laminated layers obtained through ultraviolet laser lithography. The spread layer of resin paste became thinner, according to the volume fraction of the particles after laser dewaxing and sintering.

Figure 8 shows a schematic of the layer lamination in the ultraviolet laser lithography. Through the ultraviolet laser drawing on the resin paste containing ceramic particles at a volume fraction of 50%, the laminated layer with 20 µm in spreading thickness became thinner, reaching a half pitch of 10 µm after dewaxing and sintering. The linear shrinkage of 50% was observed solely for the *Z*-axis direction, according to the volume fraction of the particle dispersion. The resin dewaxing and the powder sintering were realized via laser-beam ablation towards the spread paste without shrinkage for the *X*- and *Y*-axis directions. The reaction force of resin vaporization was considered to compress the remained particles in the direction perpendicular to the laminated layer.

**Figure 9:**
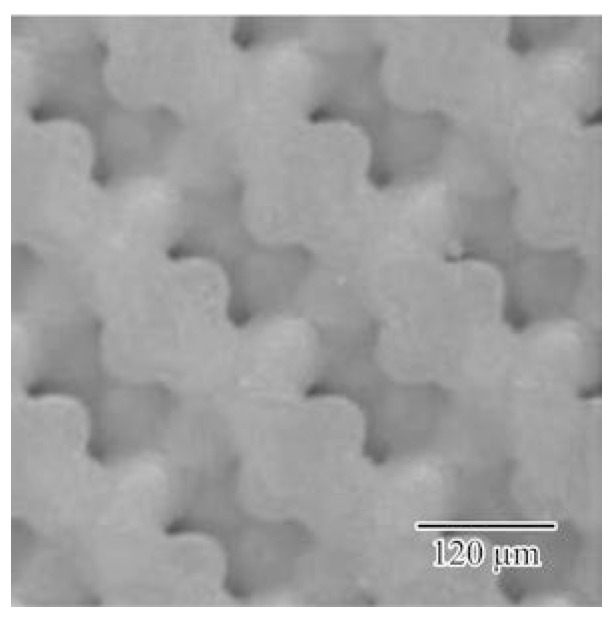
Titania photonic crystal subjected to heat treatment of post-sintering in air. The linear shrinkage due to the treatment was <1%, compared with the sample dimension after the lithographic processing.

**Figure 10:**
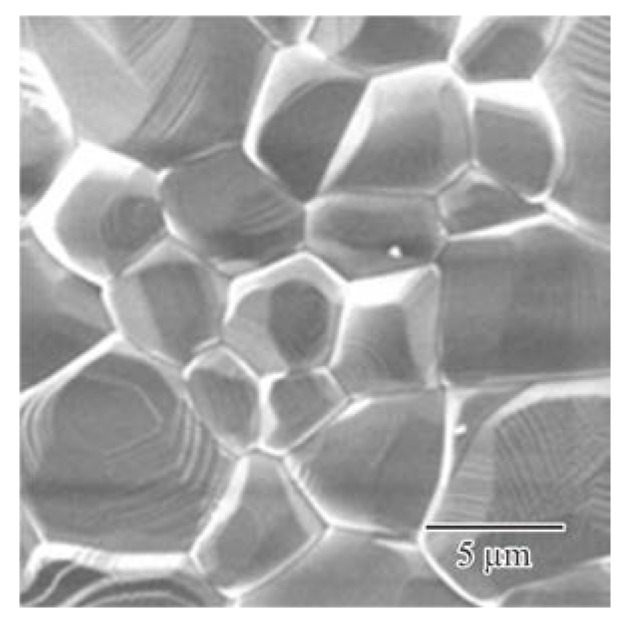
Microstructure of the titania lattice after the post-sintering, observed by SEM. The fine ceramic structure without micro-cracks or pores had a high relative density of 99%, according to the Archimedes’ method.

The titania photonic crystal formed via ultraviolet laser lithography was heated at 1350 °C for 2 h in air to transform the crystal structure to the rutile phase. Figure 9 shows a top view of the titania lattices after the heat treatment. The linear shrinkage due to the treatment was < 1%. The lattice constant of the diamond structure was 240 µm. Figure 10 shows an SEM image of the titania lattice. No cracks or pores were observed in the microstructures. The relative density of the sample reached 99.5%. The dielectric constant and the loss of the sintered titania were measured as 100 and 0.02 at 300 GHz, respectively. The THz-wave transmission spectra of the photonic crystals were measured for the Γ-X <100>, Γ-K <110>, and Γ-L <111> directions by using the THz-TDS. The forbidden bands, prohibiting the electromagnetic waves, were formed for these crystal directions, as shown in the Figure 2. The solid circles indicate the higher and lower edges of these forbidden bands. These measured results agreed well with the calculated photonic bandgaps in the electromagnetic band diagram drawn using the PWE method. The common region was opened from 0.26 THz to 0.44 THz, as the perfect photonic bandgap diffracted the electromagnetic waves effectively for all crystal directions.

**Figure 11:**
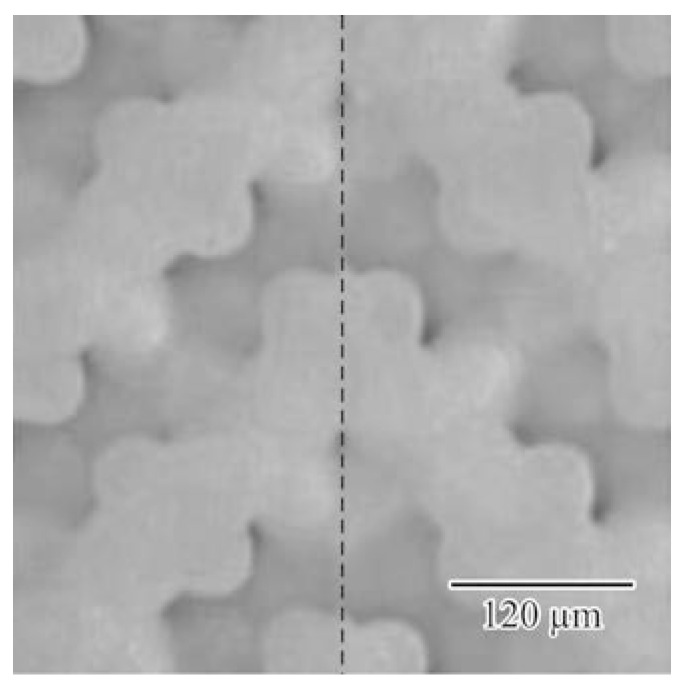
Twinned titania photonic crystal with mirror-symmetric diamond lattices. The dotted line shows an introduced stacking fault parallel to the (100) crystal plane. Two blocks of three period lattices were connected. The dotted line indicates the introduced plane defect.

**Figure 12:**
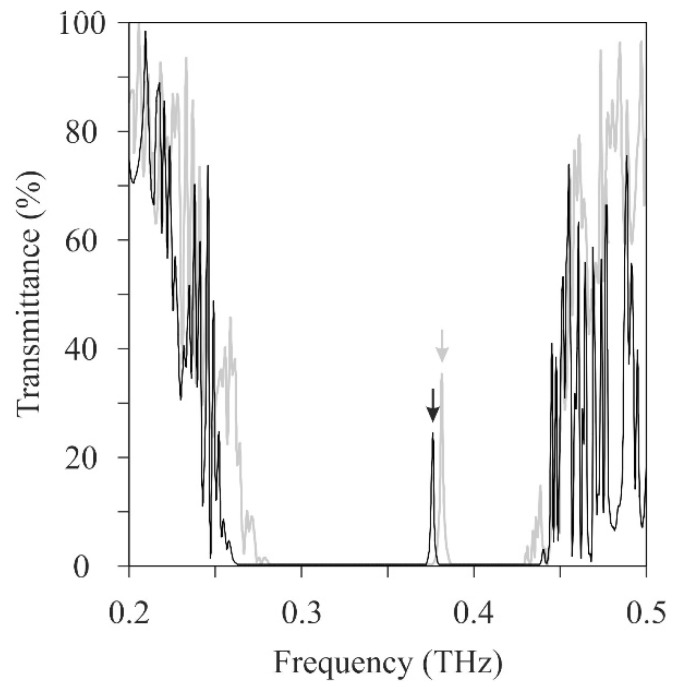
THz-wave transmission spectra of the twinned titania photonic crystal. Black and gray lines were measured using a TDS and calculated via the TLM method, respectively. Arrow heads indicate the localized mode formations in the photonic bandgaps.

**Figure 13:**
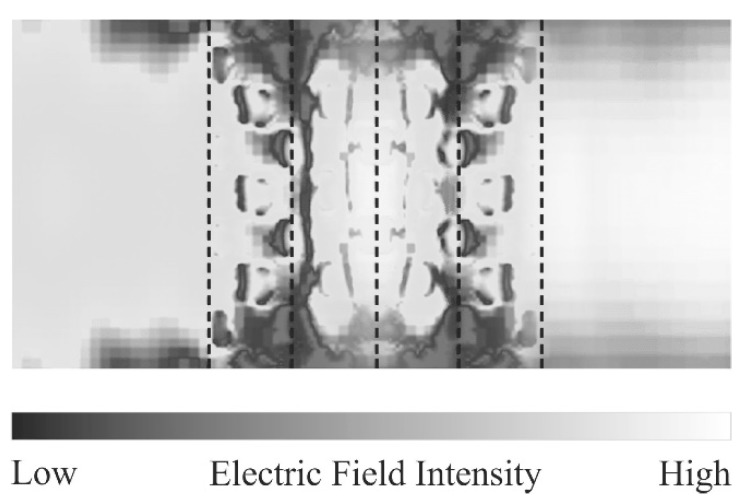
Intensity distribution of the electric field in the vicinity of a stacking fault in the twinned photonic crystal simulated at the localized mode frequency. The white and black areas of higher and lower intensities, respectively, were visualized through the TLM method.

Figure 11 shows the twinned diamond photonic crystal composed of the mirror-symmetric titania lattices. The twinned interfaces parallel to the (100) lattice plane were sandwiched between the lattice blocks with three period structures. The transmission intensities were measured using the THz-TDS and calculated via the TLM method, as shown in Figure 12. The solid and dotted lines show the measured and simulated transmission spectra, respectively. The measured results closely match the simulated ones. The measured transmittances were decreased in the conduction bands, comparing with the calculated ones by the dielectric loss of titania lattices. The localized mode peak was formed at 0.37 THz in the photonic bandgap. A quality factor or the peak was calculated at 154. Figure 13 shows a cross-sectional distribution image of the electric-field intensity in the twinned lattice structure at the resonant frequency. The white and black areas indicate regions, where the electric-field intensity was high and low, respectively, and the electromagnetic waves propagated towards the right-hand side. The incident waves were resonated and localized in the defect interface through the multiple reflections between the mirror-symmetric diffraction lattices. This amplified electromagnetic wave can be transmitted to the opposite side of the photonic crystal, and form a transmission peak in the photonic bandgap. Frequency selection and plane-wave radiation have the potential in THz-wave filter and directional antenna enabled applications.

## 5. Conclusions

Titania photonic crystals, composed of dielectric lattices with diamond structures, were developed to control THz waves by using ultraviolet laser lithography together with additive manufacturing. The microlattices were designed to exhibit perfect photonic bandgaps to prohibit electromagnetic dispersions for all crystal directions. Through laser-beam drawing and ablations on acrylic resin paste, including titania nanoparticles, solid layers with 2D cross sections were formed via dewaxing and sintering. Additionally, 3D titania components with a relative density of 97% were created successfully with a part accuracy of ±10 µm. The crystal structure of the titania lattice was transformed into the rutile phase perfectly through post-heat treatment. A perfect photonic bandgap was opened from 0.26 THz to 0.44 THz, which agreed with an electromagnetic band diagram obtained via the PWE method. A plane defect introduced between the twinned diamond lattices formed a localized mode of the transmission peak in the photonic bandgap. Plane-wave radiation at a selected frequency, through multiple reflections between the mirror-symmetric diamond lattices, was simulated and visualized using the TLM method. The twinned diamond photonic crystals of the THz wave beam generator will be applied to scanning electromagnetic microscopy for analysis and imaging of biological materials.

## Figures and Tables

**Table 1:** Process conditions in ultraviolet laser lithography.

Laser Conditions	A	B	C
Spot Size (µm)	10	10	10
Scan Speed (mm/s)	50	100	100
Irradiation Power (mW)	600	600	700
